# A Comprehensive Assessment of Apigenin as an Antiproliferative, Proapoptotic, Antiangiogenic and Immunomodulatory Phytocompound

**DOI:** 10.3390/nu11040858

**Published:** 2019-04-16

**Authors:** Alexandra Ghițu, Anja Schwiebs, Heinfried H. Radeke, Stefana Avram, Istvan Zupko, Andrea Bor, Ioana Zinuca Pavel, Cristina Adriana Dehelean, Camelia Oprean, Florina Bojin, Claudia Farcas, Codruta Soica, Oana Duicu, Corina Danciu

**Affiliations:** 1 Department of Pharmacognosy, “Victor Babeş” University of Medicine and Pharmacy, 2, Eftimie Murgu Square, 300041 Timişoara, Romania; ghitu.alexandra@gmail.com (A.G.); stefana.avram@umft.ro (S.A.); corina.danciu@umft.ro (C.D.); 2 Pharmazentrum Frankfurt/ZAFES, Institute of General Pharmacology and Toxicology, Clinic of the Goethe University, D-60590 Frankfurt/Main, Germany; schwiebs@med.uni-frankfurt.de (A.S.); radeke@em.uni-frankfurt.de (H.H.R.); 3 Department of Pharmacodynamics and Biopharmacy, University of Szeged, Eötvös u. 6, H-6720 Szeged, Hungary; zupko@pharm.u-szeged.hu (I.Z.); andrea.bor@pharm.u-szeged.hu (A.B.); 4 Department of Toxicology, “Victor Babeş” University of Medicine and Pharmacy, 2, Eftimie Murgu Square, 300041 Timişoara, Romania; cadehelean@umft.ro; 5 Department of Drug Analysis, Chemistry of the Environment and Food, “Victor Babeş” University of Medicine and Pharmacy, 2, Eftimie Murgu Square, 300041 Timişoara, Romania; camelia.oprean@umft.ro; 6 OncoGen Centre, “Pius Brînzeu” Emergency County Hospital, 156, Liviu Rebreanu Boulevard, 300736 Timişoara, Romania; florinabojin@umft.ro; 7 Department of Functional Sciences, “Victor Babeş” University of Medicine and Pharmacy, 2, Eftimie Murgu Square, 300041 Timişoara, Romania; 8 Department of Pharmaceutical Physics, “Victor Babeş” University of Medicine and Pharmacy, 2, Eftimie Murgu Square, 300041 Timişoara, Romania; farcas.claudia@umft.ro; 9 Department of Pharmaceutical Chemistry, “Victor Babeş” University of Medicine and Pharmacy, 2, Eftimie Murgu Square, 300041 Timişoara, Romania; codrutasoica@umft.ro; 10 Department of Functional Sciences—Pathophysiology, “Victor Babeş” University of Medicine and Pharmacy, 2, Eftimie Murgu Square, 300041 Timişoara, Romania; oanaduicu@umft.ro

**Keywords:** apigenin, A375 human melanoma cell line, proliferation, apoptosis, mitochondrial bioenergetics and glycolysis, angiogenesis

## Abstract

Apigenin (4′,5,7-trihydroxyflavone) (Api) is an important component of the human diet, being distributed in a wide number of fruits, vegetables and herbs with the most important sources being represented by chamomile, celery, celeriac and parsley. This study was designed for a comprehensive evaluation of Api as an antiproliferative, proapoptotic, antiangiogenic and immunomodulatory phytocompound. In the set experimental conditions, Api presents antiproliferative activity against the A375 human melanoma cell line, a G2/M arrest of the cell cycle and cytotoxic events as revealed by the lactate dehydrogenase release. Caspase 3 activity was inversely proportional to the Api tested doses, namely 30 μM and 60 μM. Phenomena of early apoptosis, late apoptosis and necrosis following incubation with Api were detected by Annexin V-PI double staining. The flavone interfered with the mitochondrial respiration by modulating both glycolytic and mitochondrial pathways for ATP production. The metabolic activity of human dendritic cells (DCs) under LPS-activation was clearly attenuated by stimulation with high concentrations of Api. Il-6 and IL-10 secretion was almost completely blocked while TNF alpha secretion was reduced by about 60%. Api elicited antiangiogenic properties in a dose-dependent manner. Both concentrations of Api influenced tumour cell growth and migration, inducing a limited tumour area inside the application ring, associated with a low number of capillaries.

## 1. Introduction

Natural products, either in the form of total extracts or purified active compounds, have been demonstrated to play a vital role in the current management of different types of cancer, with directions being pulled toward both treatment and prevention. As proven by current cancer therapy, an increased number of anticancer drugs used in the clinic are based on natural products obtained from different sources (plants, animals, microorganisms) [1]. Due to the fact that the biodiversity of our planet has not been fully exploited, many specialised research institutions, including the National Cancer Institute, have allocated important funds and brilliant minds in order to use what mother nature provided to fight the biggest challenge of the 21st century medicine: cancer.

Important studies in the field have shown that the incidence of melanoma has been growing especially in countries with light skin population [2]. Although melanoma accounts for only around 1% of the types of skin cancers, it is the most dangerous form, as it is responsible for most death cases. Moreover, the number of Americans diagnosed with skin cancer at a certain point in their lives in the last thirty years is estimated to be higher than the number of all other cancers summed up [3]. The standard models of evolution include: (a) benign naevi; (b) dysplastic naevi; (c) melanoma in situ; and (d) invasive melanoma [4]. The number of papers on PubMed that have the word “melanoma” in their title exceeds 120,000, thus indicating that melanoma represents a continuing hot topic that is being approached by an impressive number of different therapeutic strategies.

With respect to the main classes of natural compounds, flavonoids have been intensively studied as natural compounds with chemo-preventive properties against different types of cancer due to their biological activities which include antiproliferative and proapoptotic effects [5]. Apigenin (4′,5,7-trihydroxyflavone) (Api) is a natural compound belonging to the flavone subclass of flavonoids. The aglycone is part of the chemical composition of some glycosides, the main representatives being apigetrin, vitexin, isovitexin, apiin [6]. The flavone is an important component of the human diet, being distributed in a large category of fruits, vegetables and herbs, with the most important sources being represented by chamomile, celery, celeriac and parsley [7]. Other frequently used nutraceutics rich in this flavonol include oranges, grapefruit, garlic, and propolis [8]. Venigalla et al. established that in the case of ligulate flowers of chamomile, apigenin represents around 68% of the total flavonoids [9]. Apigenin has been described by numerous in vitro and in vivo studies in the field, using various cancer cell lines as a natural compound with chemo-preventive activity and tumour growth inhibition potential [10,11,12]. The relationship between cancer and inflammation is very well defined in the scholarly literature [13]. Following a complex study, Perrott et al. assigned apigenin some anti-inflammatory properties [14]. A current comprehensive review describes other biological properties of apigenin as follows: prevention of cardiovascular diseases due to different causes (atherosclerosis, hypertension, cardiac hypertrophy, induced heart injury), protective effect on the liver, on the respiratory system, on the endocrine system, on central nervous system, on bones and joins [8].

A recent paper has reported that the flavone exhibited antiproliferative, anti-invasive and proapoptotic properties in vitro against two human cancer cell lines, namely A375 and C8161 [15]. Following the same train of thought, a study conducted by Caltagirone et al. confirmed apigenin and quercetin as active compounds presenting the potential to inhibit melanoma onset and metastatic spreading in a murine model of melanoma designed by the injection of B16-BL6 cells into C57BL/6N mice [16]. This approach was investigated in more detail by the group of Piantelli et al., who assigned the anti-metastatic effect to a mechanism that involves impairing tumour cell endothelium interactions [17]. Using B16-F10 cell injection into syngeneic mice, Cao et al. proposed an additional mechanism, namely the inhibition of the STAT3 signalling pathway [18].

The aim of this study was to assess the antiproliferative, proapoptotic, antiangiogenic properties, the modulation of mitochondrial respiratory chain and the glycolysis by this flavone against A375 human melanoma cells, as well as to analyse its immunomodulatory effect in human dendritic cells.

## 2. Materials and Methods

Apigenin ≥99% (HPLC) (CAS Number 520-36-5) (Api) was acquired from Sigma-Aldrich, Steinheim, Germany.

### 2.1. Cell Culture and Preparation

The human melanoma (A375) cell line (ECACC; Sigma Aldrich origin Japan stored UK) was grown into Dulbecco’s Modified Eagle’s Medium (DMEM; Gibco BRL, Invitrogen, Carlsbad, CA, USA) supplemented with 1% penicillin/streptomycin mixture (Pen/Strep, 10,000 IU/mL; PromoCell, Heidelberg, Germany) and 10% foetal calf serum (FCS; PromoCell, Heidelberg, Germany). At 80–90% confluence, the cells were passaged following treatment with EDTA (5 mM).

Human dendritic cells were differentiated from isolated PBMCs by buffy coats, as described previously [19]. Briefly, Ficoll (GE Healthcare, Uppsala, Sweden) was used for density centrifugation.

Several (2 × 10^8^) cells per well of isolated, PBMCs were plated, and the supernatant was discarded upon 2 h of plastic adherence. Afterwards, RPMI 1640 GlutaMax medium (Thermo Fisher Scientific, Boston, MA, USA) was used for cell differentiation. The medium was supplemented with 100 IU/mL penicillin, 100 µg/mL streptomycin with 10% FCS, 10 mM HEPES (Sigma-Aldrich, Steinheim, Germany), 1 mM sodium pyruvate and 50 µM 2-β-ME (Thermo Fisher Scientific, Boston, MA, USA) supplemented with 40 ng/mL recombinant human GM-CSF (Peprotech, NJ, USA) and human IL4 (Peprotech, NJ, USA); the medium was renewed after 4 days.

Differentiated cells were collected by cell scraping and transferred to 6-well plates for further experiments or to tissue treated 8-well chambered cover slides (Ibidi, Martinsried, Germany) for fluorescence microscopy staining. The supernatants were tested for TNF-alpha IL-10 and IL-6 (R&D Systems, Wiesbaden, Germany) by ELISAs according to the manufacturer’s protocol.

### 2.2. Cell Viability Assays

The antiproliferative property of Api was determined by means of MTT (Sigma-Aldrich, Budapest, Hungary) assay against A375 human melanoma cells. Experiments were carried out as described previously [20]. Briefly, cells were seeded into 96-well plates (5000 cells/well) and treated with different concentrations of Api (0.3–60.0 µM) under standard conditions (37 °C, 5% CO_2_). After 72 h of incubation, 5 mg/mL MTT solution was added and the microplates were incubated for an additional 4 h. The resulting formazan crystals were dissolved in dimethyl sulfoxide and the absorbance was determined at 545 nm with an ELISA reader (Awareness Technology, Palm City, FL, USA). Cisplatin, a clinically used anticancer agent was applied as a reference agent. Sigmoidal concentration–response curves were fitted to the determined results and IC_50_ values were calculated by means of GraphPad Prism (GraphPad Software, San Diego, CA, USA).

For cells viability assessment, the XTT assay (Thermo Fischer Scientific) was used according to the manufacturer on human dendritic cells. Briefly, the final XTT solution was put on wells with cells or medium only as a control. After 45 min incubation time at 37 °C and 5% CO_2_, aliquots of the cells were assessed in flat 96-well plates (Greiner, Frickenhausen, Germany) at 460 and normalised to 650 nm. Previously, cells had been stimulated with the vehicle or with Api, in the presence or absence of LPS for 24 h and 48 h at the indicated concentrations. After XTT assay, the cell number was obtained and normalised to 10^4^ cells.

### 2.3. Cell Cycle Analysis by Flow Cytometry

To describe cell cycle distribution, the DNA content of the cells was determined by flow cytometry. A375 cells were plated into 6-well plates (300,000–400,000 cells/well) and pre-incubated for 24 h. Then the cells were washed twice with cold phosphate-buffered saline (PBS), trypsinised and centrifuged (1500 rpm, 10 min). Cells were washed and fixed in 1 mL of cold ice 70% ethanol for 30 min. Samples were treated with dye solution containing RNAse A (0.02 mg/mL), propidium iodide (0.1 mg/mL), Triton-X (0.003 mL/mL) and sodium citrate (1.0 mg/mL) in distilled water and the mixtures were kept in the dark for one hour at room temperature. DNA content of the cells was analysed by a Partec CyFlow flow cytometer (Partec GmbH, Münster, Germany). In each sample, 20,000 cells were assessed, and the proportion of the cells in the different cell cycle phases (subG1, G1, S and G2/M) were calculated using ModFit (Verity Software House, Topsham, ME, USA).

### 2.4. Anti-Migratory Potential—Scratch Assay Method

For the assessment of the regressive effect of Api on the invasion capacity of the human melanoma-A375 cell line, the scratch assay test was performed. Several 2 × 10^5^ cells/well were seeded onto 12-well culture plates until 90% confluence was reached. After that, the attached cells were scratched following the diameter of the well using a sterile pipette tip. The detached cells and cellular debris were removed by gently washing the wells with PBS. Furthermore, the cells were stimulated with Api at two different concentrations 30 μM and 60 μM. Wells were captured on images at 0 h and 24 h, in order to compare the cell growth of the stimulated vs. control (no stimulation) cells in early stages and at consistent times. Each well was marked below with a line, to improve identification of the same imaging area. Images were taken with Olympus IX73 inverted microscope provided with DP74 camera (Olympus, Tokyo, Japan) and cellSense Dimension software was used for analysing the cell growth. To quantify the migratory ability of the cells, the wound closure percentage was calculated as previously described [21].

### 2.5. Determination of In Situ Caspase Activity

Caspase-3 is one of the key players in the apoptotic machinery. To determine the effects of Api on the activity of caspase-3, a colorimetric assay (Sigma-Aldrich, Budapest, Hungary) was performed. Ten and 12 million cells were plated in tissue culture flasks for control and treatment condition, respectively. After 24 h of pre-incubation, the cells were treated with Api (30 uM or 60 uM) for 48 h. Then cells were sampled, counted, centrifuged, washed with PBS and re-suspended in kit lysis buffer (10^7^ cells/100 μL), and incubated on ice for 20 min. The lysate was centrifuged, and the protein concentration of the supernatant was determined (Pierce Biotechnology, Rockford, IL, USA). According to the manufacturer’s protocol, 5.0 μL portions of treated and control lysates were incubated with 10 μL selective substrate of the enzyme (acetyl-Asp-Glu-Val-Asp-*p*-nitroaniline) in a final volume of 100 μL buffer. After a night of incubation at cell culture conditions, the absorbance of *p*-nitroaniline was measured at 405 nm with an ELISA reader. The treatment-related change in the caspase activity was expressed as fold increase.

### 2.6. Annexin V—Propidium Iodide Assay

Into 6-well plates (Greiner bio-one), 5 × 10^5^ cells/well were seeded and allowed to attach to the bottom of the plate overnight. After 24 h, the cultured medium (DMEM) was thrown and a fresh one containing Api at the highest concentration (60 μM) was added. Starting from a stock solution of 10 mM Api in DMSO, successive dilutions into the medium were performed in order to obtain the final concentration of the tested compounds. As a control sample, untreated cells were used; cells incubated with DMSO were used as solvent control. After 72 h, the cells were trypsinised and analysed for the apoptotic effect of the tested compounds using flow cytometry. Annexin V-FITC mixed with propidium iodide (PI) kit (Invitrogen, ThermoFisher, Vienna, Austria) was used for cell death flow cytometric studies (apoptosis) according to the manufacturer’s manual. In brief, 2–5 × 10^5^ cells were washed two times in 1 × Annexin V binding buffer, and then centrifuged at 1,500 RPM for 5 min, re-suspended in the binding buffer and incubated with 5 μL of Annexin V-FITC in the dark for 15 min. After the cells were washed with 200 μL of the specific binding buffer and then centrifuged, the cell pellet obtained was re-suspended in a volume of 190 μL binding buffer, and immediately prior to analysis by flow cytometry 10 μL of PI solution was added.

### 2.7. Assessment of Cytotoxicity via LDH Released

Lactate dehydrogenase (LDH) assay kit (No 88954, Thermo Fisher Scientific, Boston, MA, USA) was employed to determine the cytotoxic effect of Api and DMSO at concentration of 30μM and 60 μM on the human melanoma A375 cell line. This technique is based on the cytosolic enzyme released (LDH) into the media which can be further quantified by an enzymatic reaction, leading to formazan production. The level of formazan is directly proportional to LDH leakage into the media, which is an indicative of the cytotoxic effect.

Briefly, 5000 cells/well in 200 μM specific media were cultured in 96-well plate and allowed to attach overnight. The next day, the cells were treated with Api and DMSO at concentration of 30 μM and 60 μM and incubated for 72 h. After this step, the LDH released into the media was transferred into a new 96-well plate, followed by an addition of reaction mixture. The plate was incubated at room temperature, protected from light; stop solution was added after 30 min. The concentration of formazan is measured at 490 nm and 680 nm wavelengths via spectrophotometry with a microplate reader (xMarkTMMicroplate, Serial No. 10578, Biorad, Japan).

### 2.8. Fluorescence Microscopy

Tissue-treated 8-well chambered cover slides (Ibidi, Martinsried, Germany) were used for dendritic cells growth. The cells were cultured and fixed as previously described [19]. DAPI (4′,6-Diamidine-2′-phenylindole dihydrochloride) solution (Roche Diagnostics, Mannheim, Germany) and phalloidin Alexa Flour 488 solution (Thermo Fisher) were applied for 1 h. A Zeiss LSM510 Meta system equipped with an inverted Observer Z1 microscope and a Plan-Apochromat 63 × /1.4 oil immersion objective (Carl Zeiss MicroImaging GmbH, Göttingen, Germany) were used for confocal laser scanning microscopy.

### 2.9. Extracellular Flux (XF) Analysis

A375 cells (2 × 10^4^ cells/well) were seeded in Seahorse 24-well cell culture plates and allowed to attach overnight. On the second day, the cells were stimulated with two concentrations of Api (30 and 60 µM) or with DMSO; the control group is represented by untreated cells, incubated only with cell culture medium. Background correction wells (wells that were not seeded with cells) were included in the assay, in order to normalise the data to background plate noise. Cells were incubated at 37 °C and 5% CO_2_ with the samples for 72 h.

The cells were divided into five groups: control—untreated cells; cells treated with DMSO; and cells treated with Api 30 µM and Api 60 µM, respectively. The oxygen consumption rate (OCR) and extracellular acidification rate (ECAR) were measured using the Seahorse XFe24 (Seahorse Agilent) extracellular flux analyser, as previously described [22]. Three injections were performed in order to change cells metabolism, namely: oligomycin (1 µg/mL)—a complex V inhibitor; FCCP (3 µM)—a classic uncoupling agent, and antimycin A+Rotenone (2.5 µM + 2 µM)—inhibitors of complex I and III, respectively [23]. The OCR parameters recorded in the analysis were as follows: (i) the basal respiration (before oligomycin addition); (ii) the leak state (after oligomycin addition)—the amount of O_2_ consumption needed to sustain the proton gradient; (iii) the maximal respiration (after FCCP addition)—respiration in the presence of a classic uncoupler of oxidative phosphorylation; (iv) the ATP turnover (the difference between the basal respiration and the leak state)—the amount of O_2_ consumption used for ATP production; (v) the reserve capacity (the difference between the maximal and the basal respiration). The reserve capacity is a fundamental parameter of cellular bioenergetics, and shows the capacity to respond to an increased energy demand [24]. OCR was reported in units of pmoles/minute and ECAR in mpH/minute.

### 2.10. Chorioallantoic Membrane Assay (CAM)

The CAM assay involves the use of fertilised hen (Gallus gallus domesticus) eggs. We implemented a slightly modified technique developed by Ribatti et al. [25]. Briefly, the general method includes egg disinfection with 70% ethanol prior to incubation at controlled 37 °C and 50% humidity. On the third day of incubation, noted as the embryonic day of development (EDD 3), 3–4 mL of albumen was removed, followed by cutting and resealing a window on the upper side of the eggs on EDD 4. In ovo macroscopic assessment was performed in daytime by means of a stereomicroscope (Discovery 8 Stereomicroscope, Zeiss). For further morphometric analysis, significant images were registered on a daily basis, using the Axio CAM 105 colour, Zeiss digital camera and processed by Zeiss ZEN software, ImageJ and GIMP.

The morphometric evaluation of the angiogenic reaction can be assessed using different approaches, namely semi-quantitative scales [26] or equations [27,28,29]. In this study, macroscopic images were used in order to count the number of blood vessels (BV) intersecting the inoculation ring. Angiogenesis inhibition (AI) can be expressed in percentages using the following equation:

AI (%) = (1 − No BVtest/No BVcontrol) × 100. (1)

### 2.11. Normal Angiogenesis Assessment on the Chorioallantoic Membrane

Firstly, we assessed the effects induced by Api on the normal developing CAM. This type of assay is indicative for the tolerability of Api on normal tissues, but also stands for the predictability of its implications on highly angiogenic blood vessels (between EDD 7–10). Starting on EDD 7 (day 0, 0 h) three test groups of samples were designed: (a) Api in 30 µM (API 30) concentration; (b) Api in 60 µM (API 60) concentration; (c) DMSO 1% as solvent control (1% DMSO *v*/*v* in double distilled water). All samples in volumes of 5 µL/egg were applied directly inside a plastic ring placed on top of the CAM. The assessment was carried out for 48 h, representing a medium-term tolerability assessment.

### 2.12. Tumour Angiogenesis Assessment on the Chorioallantoic Membrane

The assessment of Api in an in vivo melanoma model using the CAM assay requires the inoculation of the melanoma cells on top of the developing membrane on EDD 10 (day 0, 0 h). A375 melanoma cells were cultured according to the above described protocol and subsequently inoculated onto the CAMs [30]. Briefly, after detaching the cells from the culture plate by trypsinisation, they were cleansed and re-suspended in the culture medium until reaching the final concentration of 10^5^/5 µL. On the 10th day of incubation, 5 µL of the melanoma cell suspension was inoculated inside a plastic ring previously placed on the CAM. All specimens were inoculated with 5 µl of A375 melanoma cells and were divided in three test groups: (a) Api in 30 µM concentration; (b) Api in 60 µM concentration; (c) DMSO 1% as solvent control. Each test solution was applied in volumes of 5 µl and was repeated daily for 96 h, until EDD 14. Relevant images were captured every day in vivo, and on the final day of the experiment, after detaching the fine membranes of the tested specimens, ex vivo images were also taken. The same type of angiogenesis analysis as described for the normal tested CAMs was performed for the melanoma treated specimens.

### 2.13. Statistics

The Prism software package (Graph Pad Prism 5.0 for Windows) was used for data collection and presentation. The data ranged from three to five separate experiments is presented as the mean ± SD. An unpaired Student’s *t*-test, one-way ANOVA or two-way ANOVA followed by a Bonferroni post-test or Newman-Keuls post-test were used to determine the significant differences between the various experimental and control groups. A paired Student’s *t*-test was used to determine significant differences in all experiments concerning dendritic cells. *, **, ***, **** indicate *p* < 0.05, *p* < 0.01, *p* < 0.001 and *p* < 0.0001, respectively, compared to the control group or otherwise-indicated groups.

## 3. Results

### 3.1. Cell Growth Inhibition

**Figure 1:**
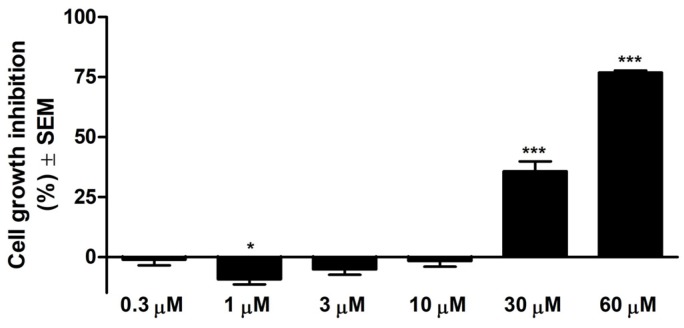
Cell growth inhibition (%) ± SEM against A375 human melanoma cells after 72 h of incubation with Api. * *p* < 0.05; *** *p* < 0.001.

As can be observed in Figure 1, in the range of tested concentrations, Api presents substantial antiproliferative effect against A375 human melanoma cell line starting from the 30 μM concentration. The calculated IC_50_ is 33.02 μM.

### 3.2. Api Effects on Cell Cycle Phases

**Figure 2:**
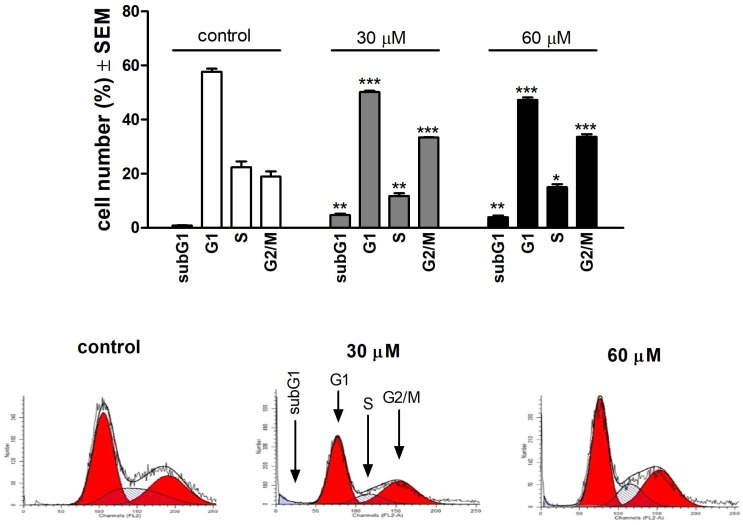
Upper panel: effects of API on the A375 human melanoma cell cycle after incubation for 24 h. Results are mean values ± SEM from three measurements. *, ** and *** indicate *p* < 0.05, *p* < 0.01 and *p* < 0.001 as compared with the control cells, respectively. Lower panel: representative histograms.

To have a complete picture of the antiproliferative effect, the concentrations that led to this kind of event, namely 30 μM and 60 μM Api, were used to analyse the effect on the phases of the cell cycle. Results showed that in the case of both concentrations, Api induced a G2/M arrest by increasing the percentage of A375 cells in this phase of the cell cycle from 18.946 ± 1.91% (control) to 33.423 ± 0.15% (30 μM Api) and 33.653 ± 0.96% (60 μM Api). Results are described in Figure 2.

### 3.3. Antiproliferative Effect of Api

**Figure 3:**
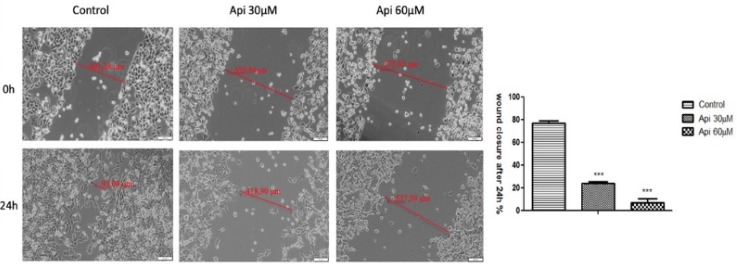
Antiproliferative effect of Api on the human melanoma A375-cell line, after stimulation with Api at 30 μΜ and 60 μΜ concentrations. Images were taken by bright field microscopy at 10× magnification. Scale bars represent 100 μm. The bar graphs are expressed as percentage of wound closure after 24 h compared to the initial surface. The data represent the mean values ± SD of three independent experiments. One-way ANOVA analysis was performed to determine the statistical differences followed by Tukey post-test (*** *p* < 0.001 vs. control—no stimulation).

As shown in Figure 3, both concentrations of Api (30 μΜ and 60 μΜ) manifested a significant inhibitory effect on the migratory capacity of human melanoma A375 cells, when compared to the migratory ability of control cells. Human melanoma A375 cells displayed wound width modifications of only 38 μm, from 575.05 μm to 537.59 μm, after treatment with Api 60 μΜ and from 550.84 μm to 418.90 μm after stimulation with Api 30 μΜ in a 24 h timeframe. The wound healing rate induced after Api treatment at 30 μΜ and 60 μΜ concentrations was 23% and 6.84%, respectively. This means that the inhibitory rate expressed by Api (30, 60 μΜ) on cell migration was 77% and 93.16%, respectively. However, the control cells (no stimulation) displayed a good wound healing rate above 77% after 24 h.

In addition, it can be observed that cells showed apoptotic characteristics by changing their shape and morphology, followed by cell detachment after 24 h, after stimulation with Api at 60 μM concentration.

The aforementioned data show that in the set experimental conditions Api exhibits antiproliferative potential.

### 3.4. Caspase 3 Activity

**Figure 4:**
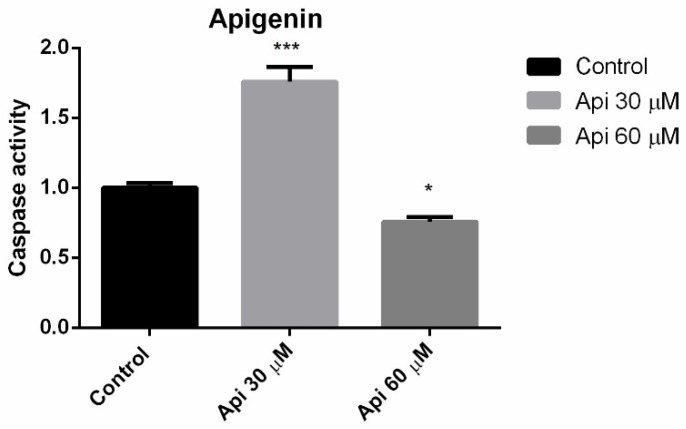
Caspase 3 activity in A375 human melanoma cells after 72 h of incubation with Api. * *p* < 0.05; *** *p* < 0.001.

A new set of experiments was conducted in order to gain insights about potential proapoptotic and/or cytotoxic effect. In this type of experiment, the Hormesis phenomena can be observed to be characterised by a biphasic response. An increased activity of protein caspase 3 following 72 h of incubation with Api 30 μM was observed. Interestingly, Api 60 μM did not enhance caspase 3 activity, presumably because of a cytotoxic effect at this concentration/incubation time (Figure 4). Also, the expression of caspase 2 and p53 proteins was analysed by immunocytochemistry, but none of these proteins were expressed following incubation with the highest tested concentration of Api (Supplementary Figure S1).

### 3.5. Annexin V-PI

**Figure 5:**
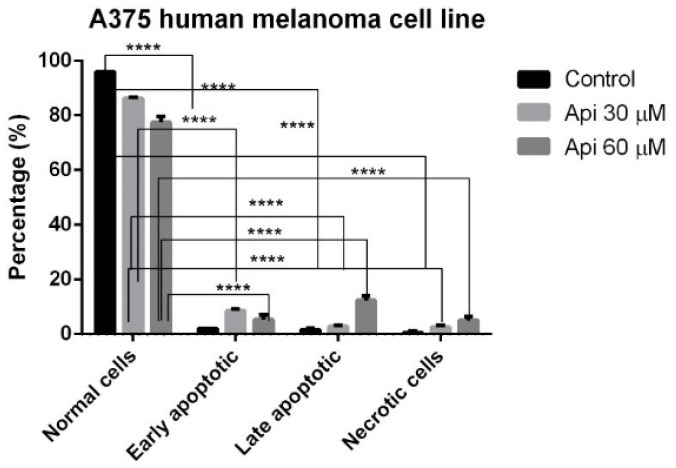
Annexin-PI staining in A375 human melanoma cells incubated with Api 30 μM and Api 60 μM, showing the populations of normal, early apoptotic, late apoptotic and necrotic cells. **** *p* < 0.0001.

This approach was followed by the Annexin-PI double staining, a consecrated assay that makes it possible to get information regarding phenomena of early apoptosis, late apoptosis and necrosis. Api at the tested concentrations (30 μM, 60 μM) induced both early and late apoptosis phenomena, as well as necrosis. As can be seen from Figure 5, where the means of three different experiments are represented, Api 30 μM caused preponderantly early apoptotic events (8.5 ± 1.8% vs. 86.25% ± 1.8 with respect to Control), while Api 60 μM (12.25 ± 2.9% vs. 77.5 ± 3.2% with respect to Control) induced predominantly late apoptotic events.

### 3.6. LDH Assay

**Figure 6:**
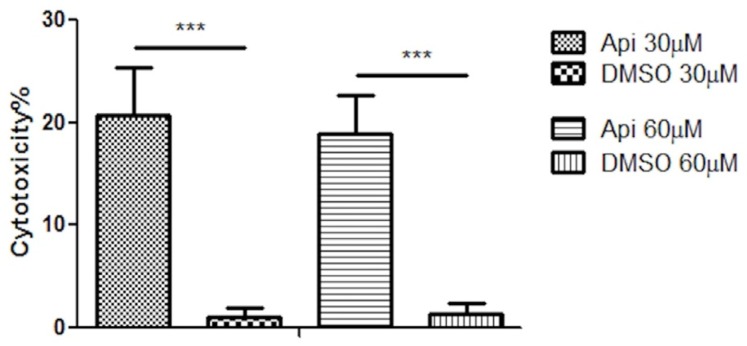
The cytotoxic effect of Api and DMSO at 30 μM and 60 μM concentrations on the human melanoma A375 cell line after 72 h exposure time. *** *p* < 0.001.

To gain more information regarding the cytotoxic potential of Api at the selected concentrations, lactate dehydrogenase assay was performed. After 72 h, a significant difference was observed in the release of lactate dehydrogenase between Api 30 μM (cytotoxicity rate of 20.75%) and DMSO (cytotoxicity rate of 1.12%). However, increment of Api concentration at 60 μM did not increase its cytotoxic effect on the human melanoma A375 cell line, showing a cytotoxic effect of almost 19%. Results are presented in Figure 6.

### 3.7. Bioenergetic Profile of A375 Human Melanoma Cells

**Figure 7:**
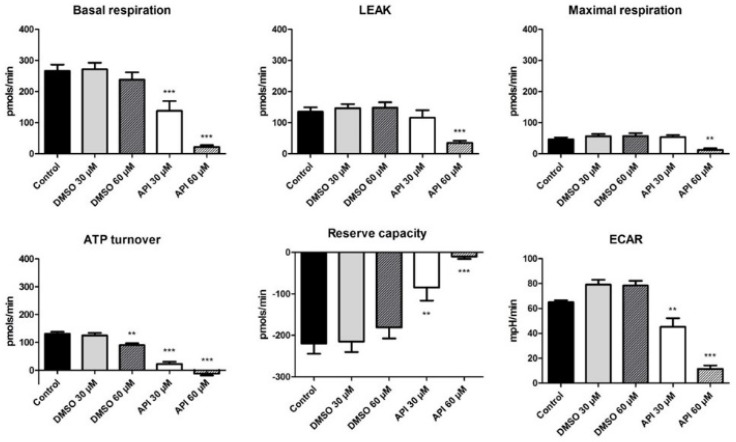
The effect of Api (30 and 60 µM) on A375 human melanoma cells (72 h treatment) with respect to OCR and ECAR parameters. ** *p* < 0.01; *** *p* < 0.001.

Within this study we also assessed the Api effect on cellular bioenergetics in A375 human melanoma cells. The oxygen consumption rate (OCR) and extracellular acidification rate (ECAR) were measured at 72 h post-treatment of A375 human melanoma cells with two concentrations of Api (30 and 60 µM) using the Seahorse XFe24 (Seahorse Agilent) extracellular flux analyser. Results describing the bioenergetic profile of A375 human melanoma cells at 72 h post-stimulation are presented in Figure 7.

We observed that in the basal respiration state, the cells are in the unchallenged state. Treatment with Api induced a significant dose-dependent decrease in the baseline rates (basal respiration for Api 30 µM → 137.9 ± 31.4 pmols/min vs. Control → 266.07 ± 20.8 pmols/min; Api 60 µM → 22.2 ± 5.5 pmols/min vs. Control). In the leakage state, there is a decrease in the OCR due to blockage of ATP production following oligomycin injection. At the higher employed dose—60 µM Api—a decrease in the proton leak state (34.9 ± 7.1 pmols/min vs. Control → 135.01 ± 14.6 pmols/min) and in the maximal respiration rate (12.4 ± 5.3 pmols/min vs. Control → 46.5 ± 5.9 pmols/min) was elicited.

After adding the respiratory chain uncoupling agent (FCCP), there is normally an increase in the O_2_ consumption due to the uncoupling mechanism. In our case, the maximal respiration was lower than the basal respiration, because in the case of tumour cells, there is a shift to a glycolytic state. The maximal respiration for Api 30 µM is 53.3 ± 6.9 pmols/min, whereas for Api 60 µM a value of 12.4 ± 5.3 pmols/min was recorded. Furthermore, the ATP turnover also displayed a decrease after stimulation with Api. This effect was recorded for Api in both tested doses and also in the case of DMSO, at the higher tested dose, 60 µM (ATP turnover for Api 30 µM was -12.65 ± 6.5 pmols/min vs. Control 131.05 ± 7.1 pmols/min and for Api 60 µM it was 22.1 ± 8.3 pmols/min vs. Control).

As previously mentioned, the reserve capacity represents the difference between the maximal and the basal respiration, and due to the fact that the first recorded lower values than the basal respiration, we obtained negative values. Our data indicate that treatment with Api decreased A375 tumour cells reserve capacity considerably, which proves that it is more difficult for treated cancer cells to respond to stress than untreated cells or cells treated with DMSO (Reserve capacity for Api 30 µM → −9.8 ± 5.8 pmols/min vs. Control → −219.5 ± 24.5 pmols/min and for Api 60 µM → −84.6 ± 31.4 pmols/min vs. Control).

In the DMSO groups, there is a slight increase in ECAR, whereas in the Api groups, there is a significant dose-dependent decrease of ECAR, indicating that the compound also induced an impairment in cellular glycolytic activity (Api 30 µM → 11.4 ± 2.8 mpH/min vs. Control → 65.04 ± 1.5 mpH/min and for Api 60 µM → 45.1 ± 7 mpH/min vs. Control). Our results indicate a significant alteration of the bioenergetic profile in A375 human melanoma cells treated with 60 µM Api, an effect that might emphasise its beneficial properties against tumour cells.

### 3.8. Immunomodulatory Effects of Api

**Figure 8:**
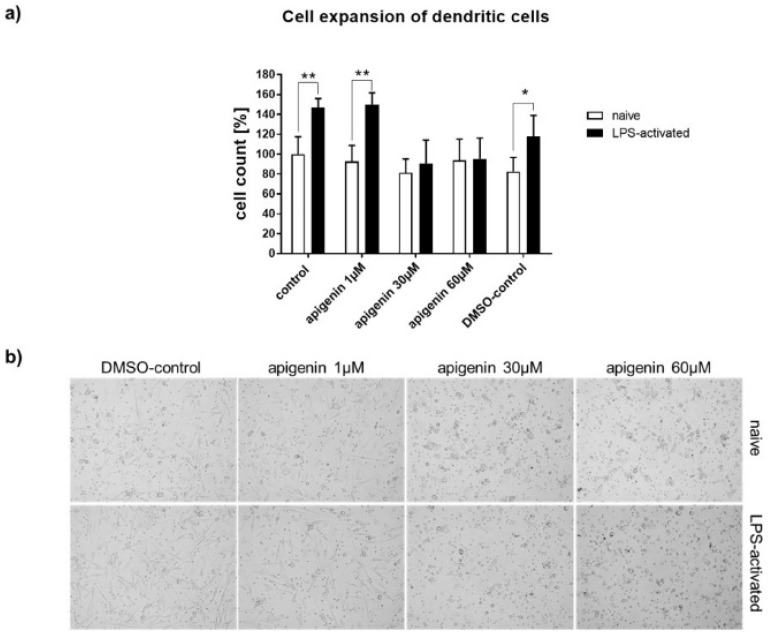
(**a**) Cell expansion of human dendritic cells after 24h with vehicle, Api in different concentrations or DMSO in the absence (native) or presence of LPS. (**b**) Representative transmitted light microscopic images of Api stimulated human dendritic cells after 24 h in the absence (native) or presence of LPS (magnification 20×). * *p* < 0.05; ** *p* < 0.01.

To test the immunomodulatory effects of Api, primary peripheral blood mononuclear cells (PBMCs) were isolated from human blood, differentiated into dendritic cells (DCs), and stimulated with corresponding amounts of Api. Cell expansion of human dendritic cells after 24 h with vehicle, Api in different concentrations, or DMSO in the absence (naïve) or presence of LPS, as well as representative transmitted light microscopic images of Api-stimulated human dendritic cells after 24 h in the absence (native) or presence of LPS, are presented in Figure 8a,b.

As expected, vehicle- and DMSO-treated cells expanded within 24 h of lipopolysaccharide (LPS) activation (Figure 8a). This cell expansion was completely abrogated by parallel stimulation of 30 and 60 µM Api, while low-dose Api (1µM) had no effect compared to control. Transmitted light microscopic images highlight the strong effect of Api on cell morphology (Figure 8b). The cells acquired a round shape upon high-dose Api stimulation. Moreover, LPS stimulation failed to provoke the typical development of dendrites as seen in the control cells or at the low dose of Api.

### 3.9. XTT Assay for Metabolic Activity

**Figure 9:**
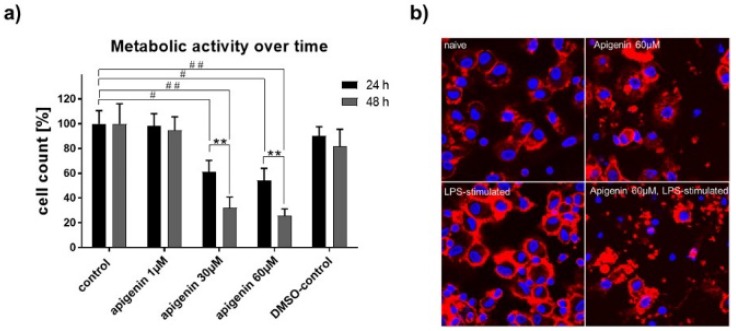
(**a**) XTT assay for metabolic activity of human dendritic cells stimulated with vehicle, Api in different concentrations or DMSO in the presence of LPS for 24 h and 48 h. (**b**) Representative confocal microscopic images of human dendritic cells treated with vehicle or 60 µM Api in absence (upper panel) or presence (lower panel) of LPS for 48 h (magnification 63x). Data are expressed as mean ± standard deviation (SD); significant differences are indicated as ** *p* < 0.01; ^##^ *p* < 0.01, ^#^ *p* < 0.05; *n* = 4.

The metabolic activity of human DCs under LPS-activation was clearly impaired by stimulation with high concentrations of Api with 24 h and even stronger within 48 h (Figure 9a). Confocal microscopy performed 48 h after stimulation revealed substantial cell damage with 60 µM Api stimulation, while control cells appeared normal (Figure 9b, upper panel). Furthermore, LPS activation led to a typical aggregation of DCs to cell-clusters under control conditions, which was not present under a high dose of Api stimulation (lower panel).

### 3.10. Cytokine Quantification

**Figure 10:**
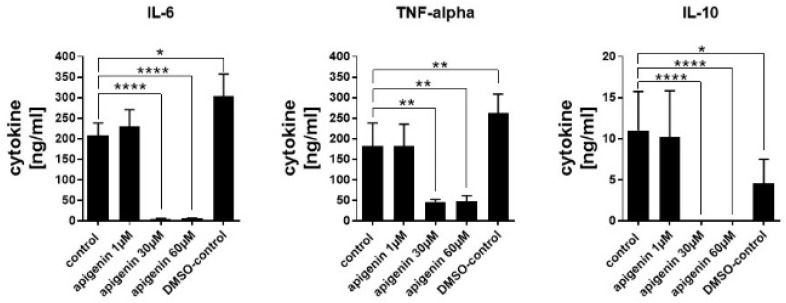
Quantification of cytokines in the supernatant of human dendritic cells stimulated with vehicle, Api in different concentrations or DMSO for 24 h in presence of LPS. Data are expressed as mean ± standard deviation (SD), significant differences are indicated as * *p* < 0.05; ** *p* < 0.01; **** *p* < 0.0001; *n* = 3.

Cytokine secretion was analysed in order to see if the reduced cell activity of LPS-stimulated cells had functional consequences. Even though DMSO led to a significant increase in IL-6 and TNF-alpha secretion, possibly because of some induced increase in the membrane permeability, the cytokine secretion was very strongly blocked by higher concentrations of Api (Figure 10). IL-6 and IL-10 secretion was almost completely blocked by 30 and 60 µM stimulation with Api and TNF alpha secretion was reduced by about 60%. The low dose of Api had no effect compared to the control secretion in all cytokines.

### 3.11. Chorioallantoic Membrane Assay

Using the in vivo chick chorioallantoic membrane assay, we investigated in ovo the tolerability and potential influence of Api on the normal and tumoural angiogenic process, next to the effect produced directly on the development of A375 melanoma cells. The assessment of Api in 30 and 60 µM concentrations was performed compared to the solvent control, diluted DMSO.

**Figure 11:**
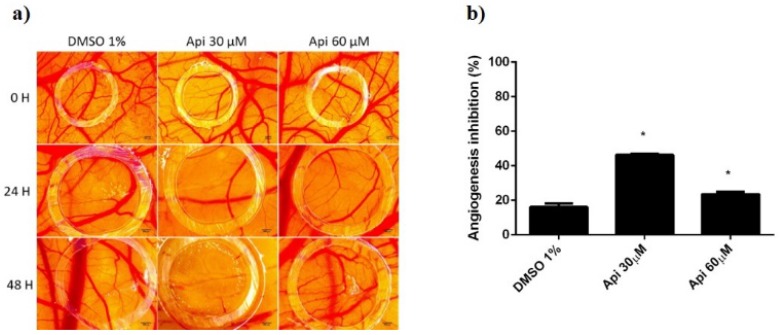
Normal CAMs treated with Api: (**a**) Stereomicroscopic in vivo images of the areas treated with Api 30 and 60 µM and with DMSO 1% as solvent control: initially—0 h, after 24 h, and after 48 h; (**b**) the angiogenic inhibition % at 48 h for Api 30 µM and 60 µM compared to DMSO 1%. * *p* < 0.05.

Firstly, we used the normal angiogenesis type of assay in order to assess the biocompatibility and tolerability. Survival rates of the embryos were similar for both concentrations of Api and the solvent control. However, some differences were observed concerning the inflammatory and irritation responses or the development of the CAM. Api in 60 µM concentration induced an irritation and fibrotic process that involved a higher number of capillaries surrounding the lesioned area. By testing the compound during a time-frame characterised by a rapid mitotic index and growth of endothelial cells, we were also able to assess the influence of Api on such a process. Interestingly, the low concentration showed a more important effect of reducing the number of capillaries inside the application area. Api at 60 µM induced a reduced inhibition of the angiogenesis, compared to Api at 30 µM, but still higher than the control (Figure 11).

**Figure 12:**
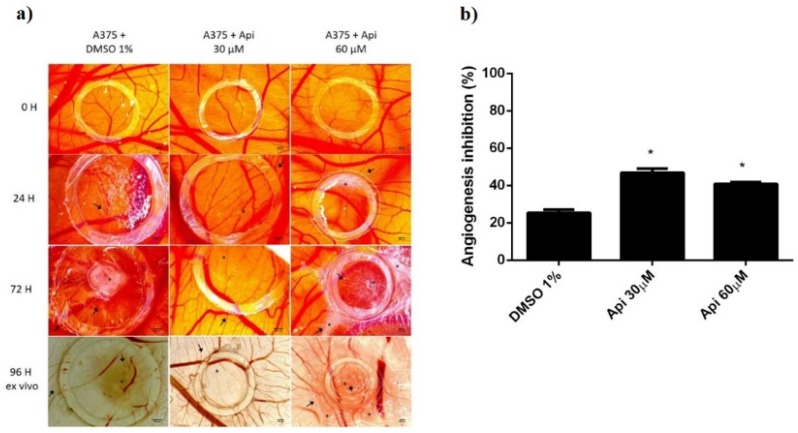
A375 melanoma cells on CAMs treated with Api: (**a**) Stereomicroscopic in vivo images of the areas previously inoculated with melanoma cells and treated with Api 30 and 60 µM and with DMSO 1% as solvent control; initially—0 h, after 24 h, after 72 h, and after 96 h, ex vivo, after membranes biopsies were obtained; (**b**) the angiogenic inhibition % in A375 melanoma cells environment, at 48 h, for Api 30 µM and 60 µM compared to DMSO 1%. * *p* < 0.05.

When tested on the tumour model in the CAM assay, using A375 melanoma cells (Figure 12), the influence of Api on tumour cells was also assessed, next to the influence of the compound on the tumour angiogenic process. Both concentrations of Api influenced tumour cell growth and migration, inducing a limited tumour area inside the application ring, associated with a low number of capillaries. Still, the migration of melanoma cells was not totally inhibited, areas of cells were observed outside the ring and spokes wheel pattern of vessels converging on the tumour cells.

Differences noticed consist of the effect induced by the two concentrations of Api. A reduced number of cells with minimal aggregation pattern were observed for the low concentration of Api (30 µM). The number of vessels inside the ring was also reduced, with capillaries showing irregularities. The higher concentration (30 µM) also showed a limited growth of tumour cells inside the ring, with reduced number of interconnected capillaries, though; the vascularisation was less inhibited compared to the low concentration, possibly owing to the irritation potential that was observed in the normal setting of CAM assay. Still, the differences between the two concentrations are reduced in the tumour angiogenesis model, and both are significantly more active in angiogenesis inhibitors compared to the control.

## 4. Discussion

In this study, we have demonstrated that Api is an antiproliferative and proapoptotic agent against the A375 human melanoma cell line, leading to an IC_50_ of 33.02 μM for the tested dose ranges (0.3–60 µM). The same conclusion was drawn by the group of Zhao et al. [15]. In their approach, in order to assess the antiproliferative potential, two melanoma cell lines were used, namely A375 and C8161, and concentrations in the interval of 40–240 µM with check points at 24 h, 48 h, 72 h and 96 h. Api inhibited proliferation in a dose-dependent manner, as well as in a time-dependent manner. Moreover, Api inhibited migration at the 40 and 80 μM concentrations after 24 h of exposure, but the effect was abolished at 100 μM. Relative to the invasion, Api inhibited this process at 40 μM concentrations after 72 h of exposure, but again the effect was abolished at 80 μM. Using a higher concentration than in our study, namely 100 μM, they also demonstrated that Api causes a G2/M arrest in the two selected melanoma cell lines. They also detected apoptotic events after 24 h of exposure at 40 and 80 μM [15]. The ability of this flavone to arrest the cell cycle in the G2/M phase in the case of epidermoid carcinoma A431 cells was postulated by Chan et al. [31]. Hasnat et al., using A375 and A2058 human melanoma cell lines, showed that Api in 50 μM concentration and after a period of incubation of 24 h significantly decreased the number and viability and altered the morphology of selected cells [32]. As discussed in the results section of our study, we have demonstrated that Api at 30 μM concentration and after a period of 72 h induced caspase 3 activation; however, when 60 μM was applied, the concentration of caspase 3 decreased as compared to control, presumably because of a cytotoxic effect at this concentration and timeframe. The assessment of the cytotoxicity was also performed by quantifying the amount of LDH, a cytosolic enzyme which is released by the cells undergoing necrosis [33,34]. However, the sensitivity of this technique is questionable when cells are exposed to compounds that induce cell cycle arrest [35]. Due to the fact that cells no longer express proliferative properties, the amount of LDH that can be released from the cells will be quite low, thus undermining the sample-induced cytotoxic effect. This phenomenon was also observed in our case for the A375 human melanoma cells stimulated with Api 60 μM. A more intense cytotoxic effect manifested by Api at 60 μM compared to the effect induced by Api at 30 μM cannot be disputed, as shown by the images performed during LDH assessment (Figure S2); yet, under these conditions, the LDH technique yielded diminished cytotoxicity results. Using the Western blot analysis, Zhao et al. showed that after 24 h of incubation, Api 100 μM augmented the expression of cleaved caspase-3 in the case of A375 and C8161 human melanoma cells [15]. In an in vivo study, Caltagirone et al. demonstrated that this flavonoid administrated intraperitoneally in mice bearing a murine model of melanoma by employing B16-BL6 cells led to a dose-dependent delay of tumour growth and decreased the number of B16-BL6 colonies in the lungs. Moreover, they showed that the phytocompound is not toxic and is able to potentiate the activity of cisplatin [16]. Along the same line of thought, Cao et al. published that the dietary flavonoid suppressed metastasis in mice bearing B16F10 lung tumours and also inhibited invasion and migration in both human and murine melanoma cell lines. A possible mechanism was assigned to the inhibition of the STAT3 signalling pathway [18]. Chao et al. showed that in the case of different human uveal melanoma cell lines, Api inhibits expression and secretion of VEGF by a mechanism that involves suppression of ERK1/2 and PI3K/Akt pathways [36]. Das et al. used Api obtained from an ethanoic extract of the plant *Lycopodium clavatum* (LC) and assessed the anticancer potential using both the A375 human melanoma cell line and the A549 human lung cancer cell line in the 20–250 µg/mL dose range interval. Their study also confirmed the in vitro antiproliferative potential against the melanoma cell line and proposed it as a mechanism for apoptosis DNA interaction, damage and mitochondrial dysfunction by the direct activity on mitochondrial oxidative phosphorylation system [37].

In the last decade, several studies have reported that Api can directly target mitochondria in tumour cell lines, revealing the activation of the mitochondrial apoptotic pathway; Api is associated with DNA fragmentation, production of reactive oxygen species, mitochondrial membrane depolarisation, release of cytochrome c and up-regulation of Bax, caspase 3, 9 and PARF [38,39,40]. Furthermore, it has been shown that Api mediates mitochondrial dysfunction in melanoma cells, namely disrupting the oxidative phosphorylation system of the A375 melanoma cell line [37]. However, the authors did not indicate whether there is impairment in the glycolytic state after Api treatment. Usually, the ECAR increase as a consequence of the cells effort to generate ATP in order to maintain their energy balance [41]. As previously mentioned, our data showed a significant alteration in the A375 human melanoma cells bioenergetic profile after the treatment with Api, especially at the higher tested dose, 60 µM, an effect that might be correlated with its beneficial effects against melanoma. To the best of our knowledge, this is the first approach that presents the effects of Api on both mitochondrial respiration and glycolysis on A375 human melanoma cells.

It is very well known that the immune system plays a crucial role with respect to development as well as resolution of malignant lesions [42]. The main ‘effectors’ for the activation of the adaptive immune system are the dendritic cells (DCs). These sentinels of the immune system have the ability to cross-present exogenous antigens to T lymphocytes [43]. On human dendritic cells, pure Api completely blocked normal LPS-mediated cell activation. Moreover, the high dose of Api even reduced typical metabolic activity of DCs. This effect was strongly reflected in a complete abrogation of the cytokine secretion of IL-6 and IL-10. We have previously shown that natural sources of Api, e.g., chamomile extracts containing about 34.103µg/100 µg extract of apigenin glucoside, or 1.388 µg/100 µg extract of apigenin, also had an effect on dendritic cell activation, although this effect was very minor compared to the pure, high dose of Api [20]. Also, the secretion of the mentioned cytokines was not reduced by chamomile extracts. Obviously, a strong concentration-dependent correlation of Api and its DC immunomodulatory effect is highlighted in the former and the current study. High doses of Api are potentially able to reduce inflammatory responses to a high extend. While Api reduces the survival of cancer cells, as indicated in this study on human melanoma cells, the effect of Api as a potential immune response suppressor has to be taken into account when considering Api as an anti-cancer agent.

Recently, Api has been studied intensively, and anticancer effects have been documented, with a possible efficacy for limiting cancer progression. Angiogenesis mediated anticancer activity is being reported in several studies, on various cancer types (e.g., lung cancer, prostate cancer, skin cancer, neuroblastoma, breast cancer) by modulating different pathways [44]. The effect was described for lung and colon cancer cells on the chorioallantoic membrane and was correlated with the decrease in the HIF-1 and VEGF expression at 20 µM concentration [45]. Our study indicated a better effect on angiogenesis inhibition in normal conditions and in the presence of melanoma cells, at 30 µM concentration, but not at 60 µM, a concentration which induced irritation phenomena on the CAM. Shankar et al. showed the effect on melanoma lung metastasis by impairing the interactions between tumour cells and the endothelial cells [44]. Little data is available on the effects of Api in vivo using the chorioallantoic membrane assay in the case of human melanoma.

## 5. Conclusions

The comprehensive assessment of Api against the A375 human melanoma cancer cell line shows that under the set experimental conditions, the flavone presents an anticancer mechanism that involves inhibition of proliferation, induction of apoptosis, modulation of bioenergetics profile and inhibition of angiogenesis. However, under the aforementioned parameters, Api does not show any immune-stimulatory properties.

## Supplementary Materials

The following are available online at https://www.mdpi.com/2072-6643/11/4/858/s1, Figure S1: Expression of caspase 2 and p53 proteins after stimulation with Api 60 µM vs. Control, Figure S2: Morphological aspects of human melanoma A375 cells exposed to Api at concentrations of 30 µM and 60 µM during LDH assessment. Images were taken 72 h post-treatment.

## References

[1] Nobili S., Lippi D., Witort E., Donnini M., Bausi L., Mini E., Capaccioli S. (2009). Natural Compounds for Cancer Treatment and Prevention. Pharmacol. Res..

[2] Hallberg Ö., Johansson O. (2013). Increasing Melanoma—Too Many Skin Cell Damages or Too Few Repairs?. Cancers.

[3] HHS. https://www.hhs.gov/.

[4] Shain A.H., Bastian B.C. (2016). From Melanocytes to Melanomas. Nat. Rev. Cancer.

[5] Chahar M.K., Sharma N., Dobhal M.P., Joshi Y.C. (2011). Flavonoids: A Versatile Source of Anticancer Drugs. Pharmacogn. Rev..

[6] Choi E.J., Kim G.H. (2009). Apigenin Induces Apoptosis through a Mitochondria/Caspase-Pathway in Human Breast Cancer MDA-MB-453 Cells. J. Clin. Biochem. Nutr..

[7] Moga M.A., Dimienescu O.G., Arvatescu C.A., Mironescu A., Dracea L., Ples L. (2016). The Role of Natural Polyphenols in the Prevention and Treatment of Cervical Cancer-An Overview. Molecules.

[8] Zhou X., Wang F., Zhou R., Song X., Xie M. (2017). Apigenin: A Current Review on its beneficial Biological Activities. J. Food Biochem..

[9] Venigalla M., Gyengesi E., Münch G. (2015). Curcumin and Apigenin—Novel and Promising Therapeutics Against Chronic Neuroinflammation in Alzheimer′s Disease. Neural Regen. Res..

[10] Sharma H., Kanwal R., Bhaskaran N., Gupta S. (2014). Plant Flavone Apigenin Binds to Nucleic Acid Bases and Reduces Oxidative DNA Damage in Prostate Epithelial Cells. PLoS ONE.

[11] Yan X., Qi M., Li P., Zhan Y., Shao H. (2017). Apigenin in Cancer Therapy: Anti-cancer Effects and Mechanisms of Action. Cell Biosci..

[12] Madunić J., Madunić I.V., Gajski G., Popić J., Garaj-Vrhovac V. (2018). Apigenin: A Dietary Flavonoid with Diverse Anticancer Properties. Cancer Lett..

[13] Rakoff-Nahoum S. (2006). Why Cancer and Inflammation?. Yale J. Biol. Med..

[14] Perrott K.M., Wiley C.D., Desprez P.Y., Campisi J. (2017). Apigenin Suppresses the Senescence-Associated Secretory Phenotype and Paracrine Effects on Breast Cancer Cells. GeroScience.

[15] Zhao G., Han X., Cheng W., Ni J., Zhang Y., Lin J., Song Z. (2017). Apigenin Inhibits Proliferation and Invasion, and Induces Apoptosis and Cell Cycle Arrest in Human Melanoma Cells. Oncol. Rep..

[16] Caltagirone S., Rossi C., Poggi A., Ranelletti F.O., Natali P.G., Brunetti M., Aiello F.B., Piantelli M. (2000). Flavonoids Apigenin and Quercetin Inhibit Melanoma Growth and Metastatic Potential. Int. J. Cancer.

[17] Piantelli M., Rossi C., Iezzi M., La Sorda R., Iacobelli S., Alberti S., Natali P.G. (2006). Flavonoids Inhibit Melanoma Lung Metastasis by Impairing Tumour Cells Endothelium Interactions. J. Cell Physiol..

[18] Cao H.H., Chu J.H., Kwan H.Y., Su T., Yu H., Cheng C.Y., Fu X.Q., Guo H., Li T., Tse A.K. (2016). Inhibition of the STAT3 signaling pathway contributes to Apigenin-Mediated Anti-Metastatic Effect in Melanoma. Sci. Rep..

[19] Schwiebs A., Thomas D., Kleuser B., Pfeilschifter J.M., Radeke H.H. (2017). Nuclear Translocation of SGPP-1 and Decrease of SGPL-1 Activity Contribute to Sphingolipid Rheostat Regulation of Inflammatory Dendritic Cells. Mediat. Inflamm..

[20] Danciu C., Zupko I., Bor A., Schwiebs A., Radeke H., Hancianu M., Cioanca O., Alexa E., Oprean C., Bojin F. (2018). Botanical Therapeutics: Phytochemical Screening and Biological Assessment of Chamomile, Parsley and Celery Extracts against A375 Human Melanoma and Dendritic Cells. Int. J. Mol. Sci..

[21] Danciu C., Muntean D., Alexa E., Farcas C., Oprean C., Zupko I., Bor A., Minda D., Proks M., Buda V. (2018). Phytochemical Characterization and Evaluation of the Antimicrobial, Antiproliferative and Proapoptotic Potential of Ephedra Alata Decne. Hydroalcoholic Extract against the MCF-7 Breast Cancer Cell Line. Molecules.

[22] Duicu O.M., Scurtu I., Popescu R., Sturza A., Coricovac D., Dănilă M.D., Privistirescu A., Muntean D.M. (2015). Assessment of the Effects of Methylene Blue on Cellular Bioenergetics in H9c2 Cells. Rev. Chim..

[23] Zuo Y.H., Han Q.B., Dong G.T., Yue R.Q., Ren X.C., Liu J.X., Liu L., Luo P., Zhou H. (2018). Panax ginseng Polysaccharide Protected H9c2 Cardiomyocyte from Hypoxia/Reoxygenation Injury through Regulating Mitochondrial Metabolism and RISK Pathway. Front. Physiol..

[24] Yadava N., Nicholls D.G. (2007). Spare Respiratory Capacity Rather than Oxidative Stress Regulates Glutamate Excitotoxicity after Partial Respiratory Inhibition of Mitochondrial Complex I with Rotenone. J. Neurosci..

[25] Ribatti D., Vacca A., Roncali L., Dammacco F. (2000). The Chick Embryo Chorioallantoic Membrane as a Model for In Vivo Research on Anti-Angiogenesis. Curr. Pharm. Biotechnol..

[26] Ribatti D. (2008). The Chick Embryo Chorioallantoic Membrane in the Study of Tumour Angiogenesis. Rom. J. Morphol. Embryol..

[27] Demir R., Peros G., Hohenberger W. (2011). Definition of the “Drug-Angiogenic-Activity-Index” that Allows the Quantification of the Positive and Negative Angiogenic Active Drugs: A Study Based on the Chorioallantoic Membrane Model. Pathol. Oncol. Res..

[28] Özcetin A., Aigner A., Bakowsky U. (2013). A Chorioallantoic Membrane Model for the Determination of Antiangiogenic Effects of Imatinib. Eur. J. Pharm. Biopharm..

[29] Zhong L., Guo X.N., Zhang X.H., Sun Q.M., Tong L.J., Wu Z.X., Luo X.M., Jiang H.L., Nan F.J., Zhang X.W. (2006). TKI-31 Inhibits Angiogenesis by Combined Suppression Signaling Pathway of VEGFR2 and PDGFRbeta. Cancer Biol. Ther..

[30] Avram S., Coricovac D.E., Pavel I.Z., Pinzaru I., Ghiulai R., Baderca F., Soica C., Muntean D., Branisteanu D.E., Spandidos D.A. (2017). Standardization of A375 human melanoma models on Chicken Embryo Chorioallantoic Membrane and Balb/c Nude Mice. Oncol. Rep..

[31] Chan L.P., Chou T.H., Ding H.Y., Chen P.R., Chiang F.Y., Kuo P.L., Liang C.H. (2012). Apigenin Induces Apoptosis via Tumour Necrosis Factor Receptor- and Bcl-2-Mediated Pathway and Enhances Susceptibility of Head and Neck Squamous Cell Carcinoma to 5-Fluorouracil and Cisplatin. Biochim. Biophys. Acta.

[32] Hasnat M.A., Pervin M., Lim J.H., Lim B.O. (2015). Apigenin Attenuates Melanoma Cell Migration by Inducing Anoikis through Integrin and Focal Adhesion Kinase Inhibition. Molecules.

[33] Chan F., Moriwaki K., De Rosa M., Snow A., Lenardo M. (2013). Detection of Necrosis by Release of Lactate Dehydrogenase Activity. Immune Homeostasis: Methods in Molecular Biology (Methods and Protocols).

[34] Renz A., Berdel W.E., Kreuter M., Belka C., Schulze-Osthoff K., Los M. (2001). Rapid Extracellular Release of Cytochrome c Is Specific for Apoptosis and Marks Cell Death In Vivo. Blood.

[35] Smith S.M., Wunder M.B., Norris D.A., Shellman Y.G. (2011). A Simple Protocol for Using a LDH-Based Cytotoxicity Assay to Assess the Effects of Death and Growth Inhibition at the Same Time. PLoS ONE.

[36] Chao S.C., Huang S.C., Hu D.N., Lin H.Y. (2013). Subtoxic Levels of Apigenin Inhibit Expression and Secretion of VEGF by Uveal Melanoma Cells via Suppression of ERK1/2 and PI3K/Akt Pathways. Evid.-Based Complement Altern. Med..

[37] Das S., Das J., Samadder A., Boujedaini N., Khuda-Bukhsh A.R. (2012). Apigenin-Induced Apoptosis in A375 and A549 Cells through Selective Action and Dysfunction of Mitochondria. Exp. Biol. Med..

[38] Chen V., Staub R.E., Baggett S., Chimmani R., Tagliaferri M., Cohen I., Shtivelman E. (2012). Identification and Analysis of the Active Phytochemicals from the anti-Cancer Botanical Extract Bezielle. PLoS ONE.

[39] Seydi E., Rasekh H.R., Salimi A., Mohsenifar Z., Pourahmad J. (2016). Selective Toxicity of Apigenin on Cancerous Hepatocytes by Directly Targeting their Mitochondria. Anticancer Agents Med. Chem..

[40] Salmani J.M.M., Zhang X.P., Jacob J.A., Chen B.A. (2017). Apigenin’s anticancer properties And Molecular Mechanisms of Action: Recent Advances and Future Prospectives. Chin. J. Nat. Med..

[41] Nicholls D.G., Darley-Usmar V.M., Wu M., Jensen P.B., Rogers G.W., Ferrick D.A. (2010). Bioenergetic Profile Experiment using C2C12 Myoblast Cells. J. Vis. Exp..

[42] Nicholson L.B. (2016). The Immune System. Essays Biochem..

[43] Spel L., Boelens J.J., Nierkens S., Boes M. (2013). Antitumor Immune Responses Mediated by Dendritic Cells: How Signals Derived from Dying Cancer Cells Drive Antigen Cross-presentation. Oncoimmunology.

[44] Shankar E., Goel A., Gupta K., Gupta S. (2017). Plant Flavone Apigenin: An Emerging Anticancer Agent. Curr. Pharmacol. Rep..

[45] Fang J., Zhou Q., Liu L.Z., Xia C., Hu X., Shi X., Jiang B.H. (2007). Apigenin Inhibits Tumour Angiogenesis through Decreasing HIF-1alpha and VEGF Expression. Carcinogenesis.

