# Supplementary material for: A Comprehensive Assessment of Apigenin as an Antiproliferative, Proapoptotic, Antiangiogenic and Immunomodulatory Phytocompound
Source: Nutrients. 2019 Apr 16;11(4):858. doi: 10.3390/nu11040858 (PMC6521017; doi:10.3390/nu11040858)
Supplement: Supplementary file 1 [file nutrients-11-00858-s001.pdf]

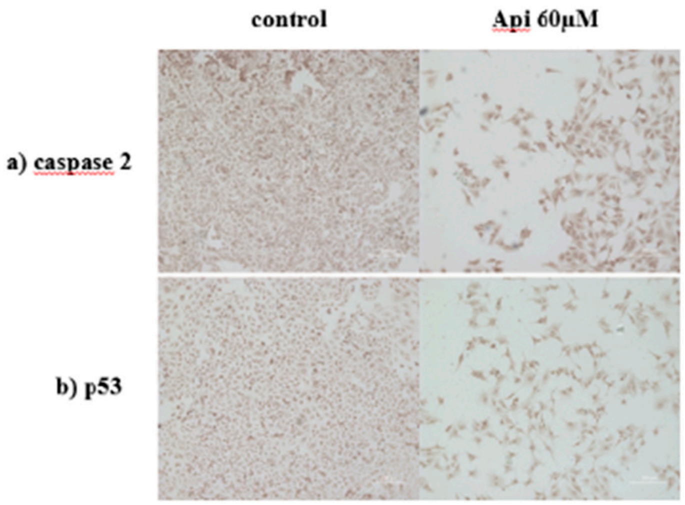

Figure S1. Expression of caspase 2 and p53 proteins after stimulation with Api 60 µM vs. Control.

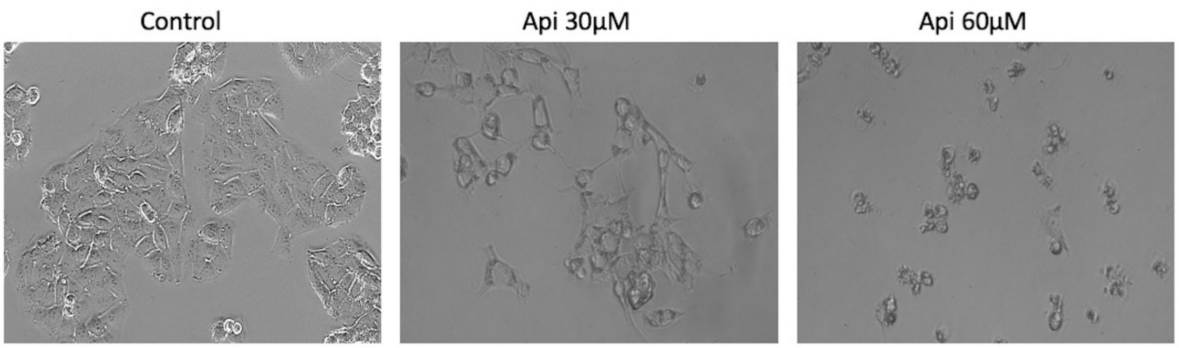

Figure S2. Morphological aspects of human melanoma A375 cells exposed to Api at concentrations of 30 µM and 60 µM during LDH assessment. Images were taken 72 h post-treatment.
